# Cancer-associated thrombosis (CAT)

**DOI:** 10.1097/HS9.0000000000000269

**Published:** 2019-06-30

**Authors:** John-Bjarne Hansen

**Affiliations:** UiT - The Arctic University of Norway, Tromsø, Norway

**Learning goals**

- Extended knowledge of pathophysiological mechanisms in cancer-related VTE may lead to targeted prevention in the future.
- Even though unprovoked VTE is associated with occult cancer, extensive cancer screening in these patients have not lead to detection of earlier stage cancers or improved prognosis.
- Recent RCTs comparing LMWHs and DOACs for treatment of cancer-related VTE have shown that DOAC treatment had less VTE recurrences and higher incidences of major bleedings. The clinical implications are uncertain.

## Introduction

Venous thromboembolism (VTE) is often the first sign of cancer, and cancer patients have a 4-7 fold higher risk of VTE compared to the general population. The VTE risk in cancer is dependent on cancer type and severity. Extended knowledge on mechanisms of cancer-related VTE would potentially lead to targeted prevention. Even though the underlying mechanisms of thrombosis risk in cancer is only partly known, tissue factor (TF), cancer cell-derived microvesicles (MV), and podoplanin, a sialoglycoprotein upregulated in several cancer cells able to induce platelet activation, are important actors in the pathophysiology of cancer-related VTE. As 5% to 10% of unprovoked VTE events are diagnosed with cancer during the following year, it has been assumed that extensive cancer screening would lead to earlier cancer diagnosis and improved prognosis. However, many studies have failed to show that extensive screening leads to detection of earlier stage cancers and improved cancer prognosis. Furthermore, clinical scores have been developed, but are not yet validated for clinical practice. Currently, guidelines recommend LMWHs for treatment of cancer-related VTE. Two independent RCTs have recently reported less VTE recurrences and higher incidences of major bleeding by DOACs compared to LMWHs for treatment of cancer-related VTE. The clinical implications are uncertain.

